# Evaluating the efficacy of laparoscopic radical antegrade modular pancreatosplenectomy in selected early-stage left-sided pancreatic cancer: a propensity score matching study

**DOI:** 10.1007/s00464-024-10868-x

**Published:** 2024-05-15

**Authors:** Zheng Li, Wenyan Xu, Ting Wang, Borui Li, Chen Chen, Yihua Shi, Chenjie Zhou, Qifeng Zhuo, Shunrong Ji, Wensheng Liu, Xianjun Yu, Xiaowu Xu

**Affiliations:** 1 Department of Pancreatic Surgery, Fudan University Shanghai Cancer Center, 270 DongAn Road, Shanghai, 200032 China; 2 Department of Oncology, Shanghai Medical College, Fudan University, Shanghai, 200032 China; 3 Shanghai Pancreatic Cancer Institute, Shanghai, 200032 China; 4 Pancreatic Cancer Institute, Fudan University, Shanghai, 200032 China

**Keywords:** Left-sided pancreatic cancer, Early-stage tumor, Laparoscopic radical antegrade modular pancreatosplenectomy, Laparoscopic distal pancreatosplecnectomy, Surgical complication, Oncologic prognosis

## Abstract

**Background:**

Laparoscopic radical pancreatectomy is safe and beneficial for recectable pancreatic cancer, but the extent of resection for early-stage tumors remains controversial.

**Methods:**

Consecutive patients with left-sided pancreatic cancer who underwent either laparoscopic radical antegrade modular pancreatosplenectomy (LRAMPS, n = 54) or laparoscopic distal pancreatosplecnectomy (LDP, n = 131) between October 2020 and December 2022 were reviewed. The preoperative radiological selection criteria were as follows: (1) tumor diameter ≤ 4 cm; (2) located ≥ 1 cm from the celiac trunk; (3) didn’t invade the fascial layer behind the pancreas.

**Results:**

After 1:1 propensity score matching (LRAMPS, n = 54; LDP, n = 54), baseline data were well-balanced with no differences. LRAMPS resulted in longer operation time (240.5 vs. 219.0 min, P = 0.020) and higher intraoperative bleeding volume (200 vs. 150 mL, P = 0.001) compared to LDP. Although LRAMPS harvested more lymph nodes (16 vs. 13, P = 0.008), there were no statistically significant differences in lymph node positivity rate (35.2% vs. 33.3%), R0 pancreatic transection margin (94.4% vs. 96.3%), and retroperitoneal margin (83.3% vs. 87.0%) rate. Postoperative complications did not significantly differ between the two groups. However, LRAMPS was associated with increased drainage volume (85.0 vs. 40.0 mL, P = 0.001), longer time to recover semi-liquid diet compared to LDP (5 vs. 4 days, P < 0.001) and increased daily bowel movement frequency. Tumor recurrence pattern and recurrence-free survival were comparable between the two groups, but the adjuvant chemotherapy regimens varied, and the completion rate of the 6-month intravenous chemotherapy was lower in the LRAMPS group compared to the LDP group (51.9% vs. 75.9%, P = 0.016).

**Conclusions:**

LRAMPS did not provide oncological benefits over LDP for left-sided pancreatic cancer within the selection criteria, but it increased operation time, intraoperative bleeding, and postoperative bowel movement frequency. These factors impacted the regimen selection and completion of adjuvant chemotherapy, consequently compromising the potential benefits of LRAMPS in achieving better local control.

**Graphical Abstract:**

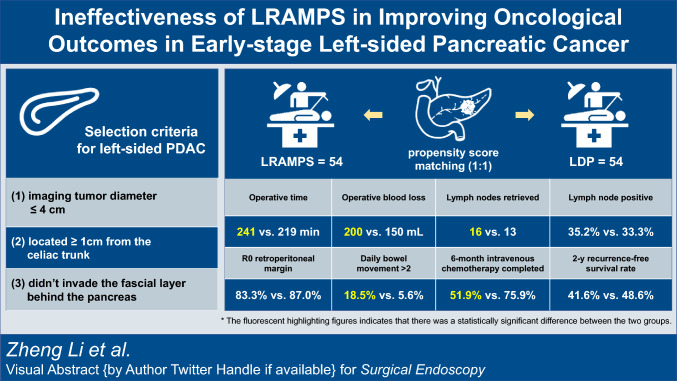

**Supplementary Information:**

The online version contains supplementary material available at 10.1007/s00464-024-10868-x.

## Introduction

For benign and low-grade malignant tumors, laparoscopic distal pancreatectomy is recommended [1]. The Miami International Evidence-Based Guidelines on Minimally Invasive Pancreas Resection also consider laparoscopic distal pancreatosplenectomy (LDP) to be feasible, safe, and oncologically equivalent for the treatment of pancreatic ductal adenocarcinoma (PDAC) [2, 3]. The DIPLOMA trial further confirmed the non-inferiority of minimally invasive surgery compared to open surgery in terms of radical resection rates for resectable left-sided PDAC [4].

However, the extent of posterior resection and the oncological safety of achieving complete N1 lymph node resection in LDP remain uncertain [3]. Strasberg proposed radical antegrade modular pancreatosplenectomy (RAMPS) as a treatment approach for resectable left-sided PDAC and confirmed its ability to achieve negative margins and satisfactory survival rates in a significant proportion of patients [5–7]. Moreover, RAMPS has demonstrated oncological advantages in terms of postoperative local control [8]. In comparison to open RAMPS, laparoscopic RAMPS (LRAMPS) not only provides similar short-term postoperative outcomes but also offers the advantages of minimally invasive surgery [9]. Further studies have also confirmed the technical feasibility of LRAMPS and its comparable long-term oncological outcomes to the open approach [10–12].

Several studies have evaluated the feasibility of LRAMPS as the standard treatment for resectable left-sided PDAC and have recommended its routine use [13–15]. However, previous studies on LRAMPS have predominantly included tumors staged T2 and above, and there is currently a lack of research on the routine use of LRAMPS for early-stage tumors. The study aims to compare the perioperative and oncological outcomes of patients with early-stage left-sided PDAC and who underwent LRAMPS or LDP using propensity score matching (PSM) process.

## Methods

### Study population and selection criteria

Consecutive patients with pathologically diagnosed left-sided PDAC who underwent laparoscopic radical pancreatectomy between October 2020 and December 2022 at Fudan University Shanghai Cancer Center were prospectively collected and retrospectively analyzed. The preoperative radiological selection criteria were as follows: (1) tumor diameter ≤ 4 cm; (2) located ≥ 1 cm from the celiac trunk; (3) didn’t invade the fascial layer behind the pancreas.

The study was approved by the Shanghai Cancer Center Institutional Review Board, and informed consent was obtained from all patients for the use of their clinical data.

### Preoperative investigations and surgical procedures

Abdominal contrast-enhanced computed tomography examination was routinely performed before operation. Selective examination of contrast-enhanced magnetic resonance imaging or positron emission tomography scan was conducted to identify potential liver or other distant metastases.

**Fig. 1:**
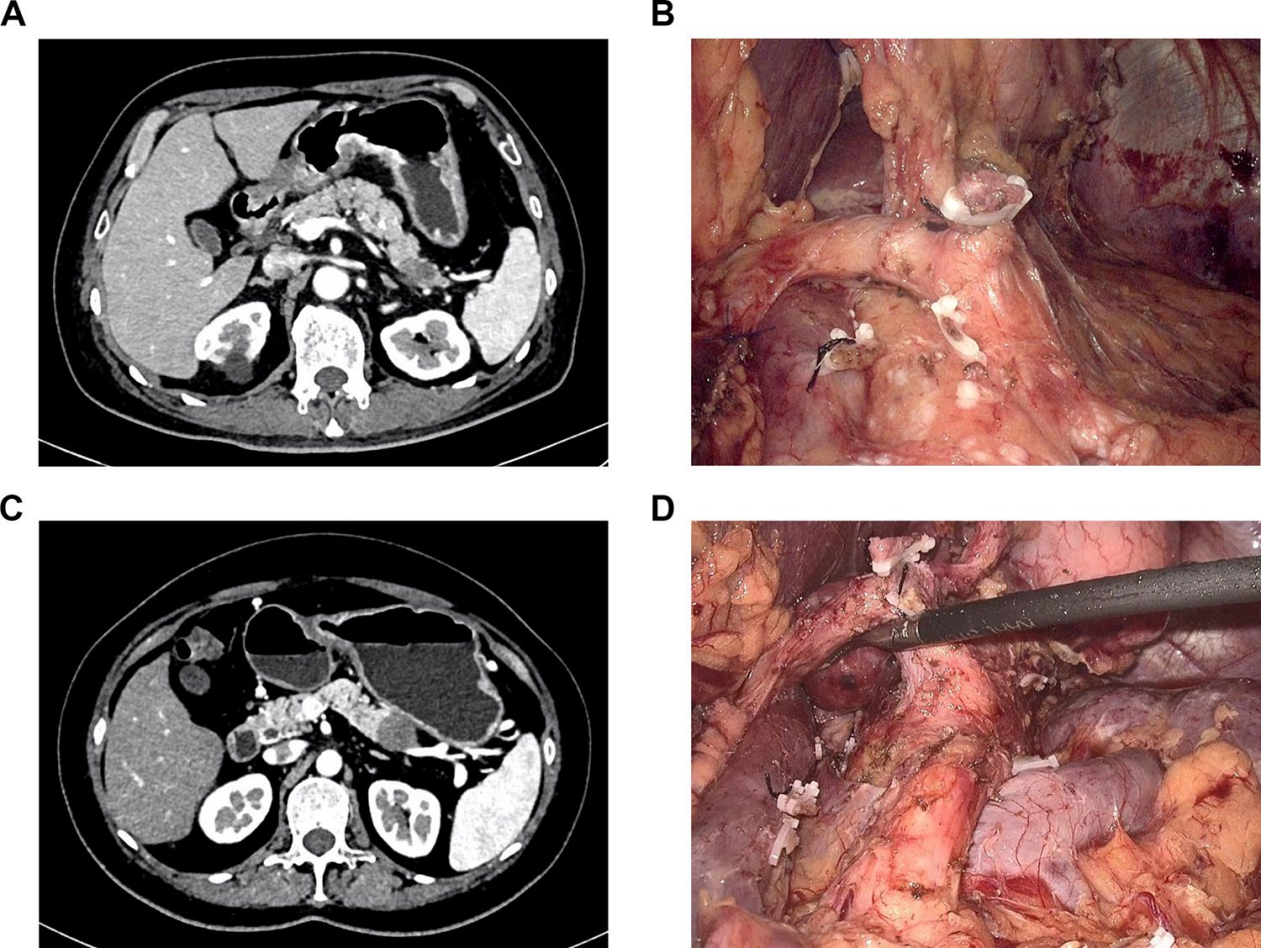
Preoperative CT images of left-sided PDAC meeting the selection criteria and the extent of surgical resection under laparoscopy. **A** and **B** Preoperative CT images (**A**) of a pancreatic tail cancer meeting the selection criteria and the corresponding surgical field after laparoscopic distal pancreatectomy (**B**); **C** and **D**: Preoperative CT images (**C**) of a pancreatic tail cancer meeting the selection criteria and the corresponding surgical field after laparoscopic radical antegrade modular pancreatosplenectomy (**D**). *CT* Computed tomography, *PDAC* pancreatic ductal adenocarcinoma

Before surgery, routine laparoscopic exploration was performed to rule out any metastases. The surgical procedure and extent of resection in LRAMPS primarily followed the guidance provided by Strasberg et al. [5–7]. In addition, the choice of approach, be it the conventional method or previously reported techniques such as the ligament of Treitz approach or the "Plane first" approach, was made based on the specific anatomical characteristics of the tumor [12, 16, 17]. In the LDP procedure, the dissection plane consistently remains located behind the fusion fascia and is not mixed. In the LRAMPS procedure, Gerota’s fascia and the perirenal fat capsule are removed. The LRAMPS procedure can be divided into anterior LRAMPS and posterior LRAMPS, depending on whether the left adrenal gland is resected. Figure 1 depicts the preoperative CT images of pancreatic tail cancer that met the selection criteria, as well as the corresponding surgical field after performing either LDP or posterior LRAMPS.

In the LDP group, the extent of standard lymphadenectomy followed the definition set by the International Study Group of Pancreatic Surgery, which includes stations 10, 11, and 18. Additionally, in cases where the tumor was situated close to the pancreatic body, the station 9 was also removed [18]. As per the definition by Strasberg et al., the LRAMPS procedure aims to achieve complete N1 lymph node dissection based on the established anatomy of lymph node drainage [5–7]. In addition to the standard lymphadenectomy, LRAMPS involves the routine clearance of stations 7, 8, 9, and 14 a/b.

In this study, the determination of surgical approach was primarily based on detailed surgical records and validated through postoperative pathological examination. All three operating surgeons in this study had over 100 cases of LDP experience, with each surgeon having performed at least 25 LRAMPS procedures and surpassed their own learning curve for LRAMPS [2, 4, 19].

### Main outcome measures and follow-up

The operation time, intraoperative blood loss, and transfusion status were meticulously recorded. Postoperative pathological examination encompassed tumor diameter, the number of harvested lymph nodes and positive lymph nodes, tumor differentiation degree, intravascular tumor thrombus, perineural invasion, and invasion of adjacent tissues. For left-sided PDAC cases meeting the selection criteria, the resection margins assessed in this study were the pancreatic transection margin and the retroperitoneal margin (i.e., posterior tangential margin). R0 margins were defined as the absence of tumor cells within a tissue distance of ≤ 1 mm from the margin, as observed under a microscope [20]. Intraoperatively, routine frozen sections of the transection margin were performed. In the event of a positive result, extended resections would be required. Tumor staging was determined according to the eighth edition TNM staging system of the American Joint Committee on Cancer.

Postoperative complications were assessed using the Clavien‒Dindo classification system [21]. The definition of postoperative pancreatic fistula (POPF), chyle leak, postpancreatectomy hemorrhage, and delayed gastric emptying followed the guidelines established by the International Study Group for Pancreatic Surgery [22–24]. The time to transition to a semi-liquid diet, removal of all abdominal drainage tubes, postoperative transfusions, reoperation, readmission, and perioperative mortality were all carefully documented. Perioperative mortality was classified as any death occurring within 90 days after the initial surgery, irrespective of the cause. If the patient’s physical condition allowed, adjuvant chemotherapy was administered 1–2 months post-surgery, and detailed records of the information were meticulously maintained.

Patient follow-up after discharge was carried out by a specialized member of the surgical team through outpatient visits or telephone. Within the first year after surgery, patients underwent chest and abdominal contrast-enhanced CT scans and tumor marker tests every 3 months, and every 6 months thereafter. If necessary, selective contrast-enhanced magnetic resonance imaging or positron emission tomography scans were performed to clarify unclear CT scan results. Local recurrence was defined as progression in the surgical area or soft tissue around the pancreatic vessels, as well as progression in the retroperitoneal lymph nodes. Early recurrence was defined as the occurrence of recurrence within 1 year after surgery, as previous studies have shown that a 1-year disease-free interval is the optimal threshold for distinguishing early and late recurrence after resection of resectable PDAC [25]. Recurrence-free survival (RFS) was defined as the duration from surgery to the occurrence of tumor recurrence, patient death, or the last follow-up.

### Propensity score matching and statistical analysis

Logistic regression analysis (Supplementary Table 1) was employed to identify potential confounding variables that could impact group allocation. Based on the results, tumor diameter on imaging and tumor differentiation were selected as covariates for the matching process. A matching ratio of 1:1 was implemented, utilizing the nearest-neighbor matching method.

Categorical variables were compared using the χ^2^ test or Fisher’s exact test. Continuous variables or variables with an abnormal distribution were compared using unpaired Student’s t test or the Mann‒Whitney U test. RFS was estimated using the Kaplan–Meier method and compared using the log-rank test. All analyses were performed using SPSS Statistics 26.0, and R 4.3.0 with the tidyverse and the MatchIt package loaded. Two-sided P < 0.05 was considered statistically significant.

## Results

### Baseline characteristics

**Fig. 2:**
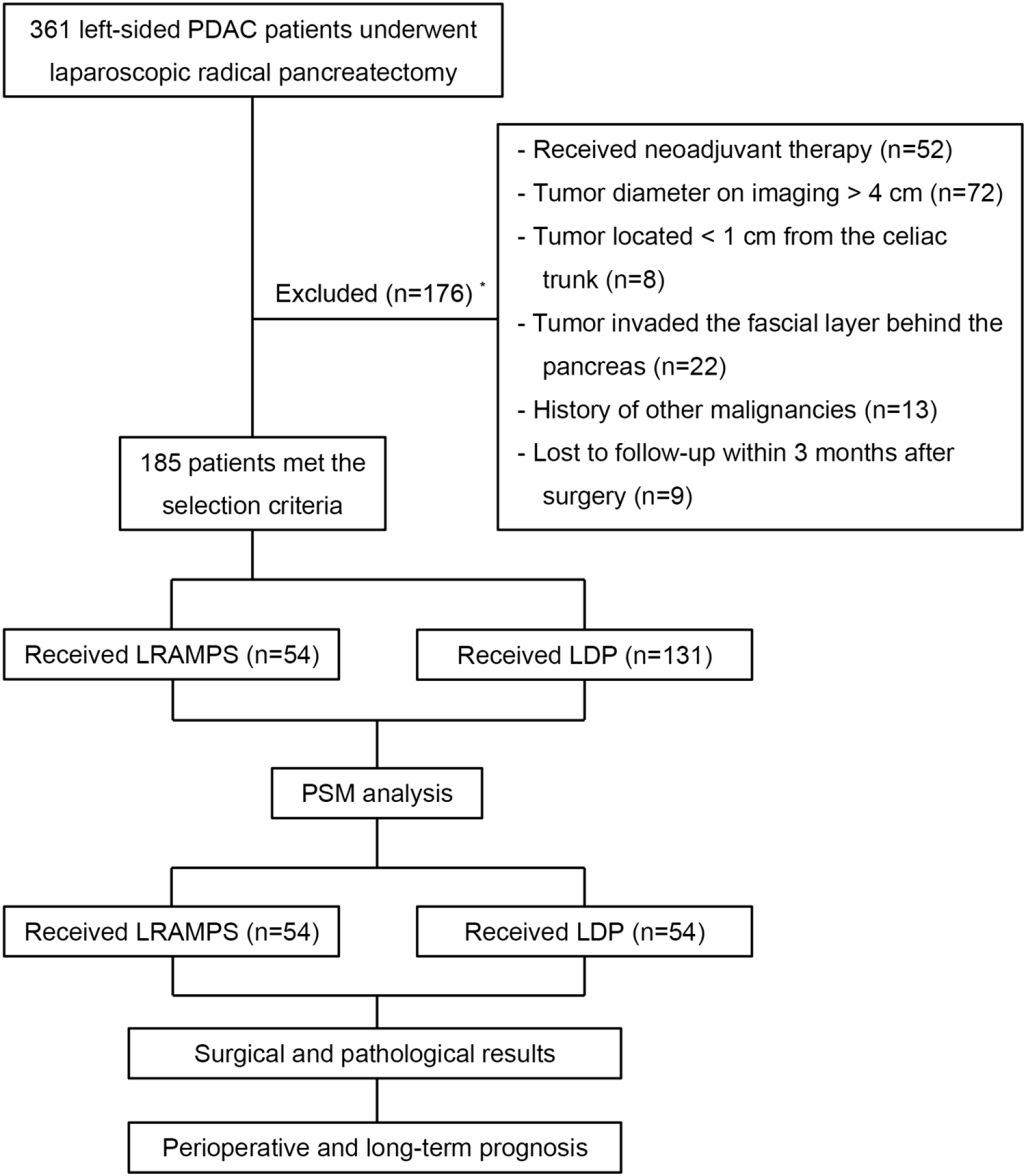
Flow chart of the study. *PDAC* Pancreatic ductal adenocarcinoma, *LRAMPS* laparoscopic radical antegrade modular pancreatosplenectomy, *LDP* laparoscopic distal pancreatosplecnectomy. *: only the main reason for exclusion is presented

The flow chart of the study is presented in Fig. 2. Three hundred and sixty-one consecutive patients were reviewed and a total of 176 patients were excluded: (1) received neoadjuvant therapy (n = 52); (2) imaging tumor diameter > 4 cm (n = 72); (3) tumor located < 1 cm from the celiac trunk (n = 8); (4) tumor invaded the fascial layer behind the pancreas (n = 22); (5) history of other malignancies (n = 13); (6) lost to follow-up within 3 months after surgery (n = 9). The remaining 185 patients met the selection criteria were included in the analysis, with 54 patients in the LRAMPS group (15 patients had anterior LRAMPS procedure and 39 posterior LRAMPS procedure) and 131 patients in the LDP group (Table 1).

**Table 1:** Baseline characteristics in the whole cohort and the PSM cohort

Variable	Whole cohort (n = 185)			PSM cohort (n = 108)		
	LDP (n = 131)	LRAMPS (n = 54)	P value	LDP (n = 54)	LRAMPS (n = 54)	P value
Age, years	66.6 (60.4–71.1)	67.0 (59.4–72.1)	0.982	67.2 (57.8–71.3)	67.0 (59.4–72.1)	0.768
Gender, male	78 (59.5)	31 (57.4)	0.788	28 (51.9)	31 (57.4)	0.562
Body mass index, kg/m^2^	23.3 (22.0–25.4)	23.3 (21.4–25.0)	0.514	23.3 (21.8–25.7)	23.3 (21.4–25.0)	0.456
Diabetes mellitus, yes	28 (21.4)	16 (29.6)	0.231	13 (24.1)	16 (29.6)	0.515
Hypertension, yes	60 (45.8)	19 (35.2)	0.184	25 (46.3)	19 (35.2)	0.240
History of abdominal surgery, yes	32 (24.4)	11 (20.4)	0.553	11 (20.4)	11 (20.4)	1.000
White blood cell, 10^^^9/L	6.3 (5.2–7.7)	6.4 (4.7–7.9)	0.990	6.3 (5.3–7.7)	6.4 (4.7–7.9)	0.705
Hemoglobin, g/L	133.0 (124.0–145.0)	137.5 (124.5–145.8)	0.780	131.0 (124.0–142.0)	137.5 (124.5–145.8)	0.269
Albumin, g/L	45.5 (42.8–47.5)	45.2 (43.1–46.8)	0.371	45.7 (43.2–48.2)	45.2 (43.1–46.8)	0.218
Total bilirubin, μmol/L	10.8 (7.9–15.2)	10.4 (7.9–15.8)	0.989	10.3 (8.1–15.3)	10.4 (7.9–15.8)	0.922
Serum creatinine, μmol/L	66.0 (56.0–78.0)	62.5 (57.5–72.0)	0.362	65.0 (54.0–75.3)	62.5 (57.5–72.0)	0.766
Prothrombin time, s	12.3 (11.5–12.8)	12.5 (11.4–13.1)	0.212	12.1 (11.4–12.6)	12.5 (11.4–13.1)	0.051
CA19-9, U/mL	128.0 (39.1–356.0)	103.6 (53.8–226.0)	0.491	169.5 (43.0–501.0)	103.6 (53.8–226.0)	0.115
Tumor diameter on imaging, cm	2.7 (2–3.3)	3.0 (2.2–3.6)	**0.047**	3.0 (2.3–3.6)	3.0 (2.2–3.6)	0.837
Tumor differentiation			**0.010**			0.119
Moderate or well	85 (64.9)	24 (44.4)		26 (48.1)	24 (44.4)	
Poor	46 (35.1)	30 (55.6)		28 (51.9)	30 (55.6)	

*PSM* Propensity score matching, *LDP* laparoscopic distal pancreatosplecnectomy, *LRAMPS* laparoscopic radical antegrade modular pancreatosplenectomy, CA19-9 carbohydrate antigens 19-9

The results are described using number (%)/median (interquartile range), and statistically significant values (P < 0.05) are given in bold

Although the selection criteria require a tumor diameter of no more than 4 cm, the LRAMPS group exhibited a larger median radiological tumor diameter than the LDP group (3.0 vs. 2.7 cm, P = 0.047). Additionally, postoperative pathology indicated a higher proportion of tumors with poor differentiation in the LRAMPS group compared to the LDP group (55.6% vs. 35.1%, P = 0.010). To address this imbalance, a 1:1 PSM analysis was conducted, resulting in a well-balanced cohort of 54 patients in each group, with no statistically significant differences observed in baseline data. All subsequent analyses were performed based on this matched cohort.

### Surgical information and pathological examination

The surgical and postoperative pathological information is presented in Table 2. The LRAMPS group exhibited a longer operative time (240.5 vs. 219.0 min, P = 0.020) and greater intraoperative blood loss (200 vs. 150 mL, P = 0.001) compared to the LDP group. The median pathological tumor diameter did not show a significant difference between the two groups (3.0 cm vs. 3.0 cm). Moreover, there was no disparity in tumor stage between the LRAMPS and LDP groups, with the proportions of T1 stage and T2 stage being 11.1% and 88.9%, and 13.0% and 87.0%, respectively.

**Table 2:** Surgical and pathological characteristics in the PSM cohort

Variable	LDP (n = 54)	LRAMPS (n = 54)	P value
ASA grade			0.437
Grade I	5 (9.3)	2 (3.7)	
Grade II	49 (90.7)	52 (96.3)	
Operative time, min	219.0 (179.5–246.8)	240.5 (203.3–274.3)	**0.020**
Operative blood loss, mL	150 (100–200)	200 (137.5–300)	**0.001**
Operative transfusion, yes	3 (5.6)	1 (1.9)	0.618
Tumor diameter, cm	3.0 (2.5–3.7)	3.0 (2.8–4.0)	0.611
Tumor stage			0.767
T1	7 (13.0)	6 (11.1)	
T2	47 (87.0)	48 (88.9)	
Lymph nodes retrieved	13.0 (10.8–16.3)	16.0 (12.8–19.3)	**0.008**
Lymph node positive, yes	18 (33.3)	19 (35.2)	0.839
Lymph node stage			1.000
N0	36 (66.7)	35 (64.8)	
N1	16 (29.6)	17 (31.5)	
N2	2 (3.7)	2 (3.7)	
AJCC 8th TNM stage			1.000
I	36 (66.7)	35 (64.8)	
II	16 (29.6)	17 (31.5)	
III	2 (3.7)	2 (3.7)	
Intravascular tumor thrombus, yes	22 (40.7)	20 (37.0)	0.693
Perineural invasion, yes	46 (85.2)	49 (90.7)	0.375
Adjacent tissue invasion, yes	43 (79.6)	42 (77.8)	0.814
R0 transection margin, yes	52 (96.3)	51 (94.4)	1.000
R0 retroperitoneal margin, yes	47 (87.0)	45 (83.3)	0.588

*PSM* Propensity score matching, *LDP* laparoscopic distal pancreatosplecnectomy, *LRAMPS* laparoscopic radical antegrade modular pancreatosplenectomy, *ASA* American Society of Anesthesiologists, *AJCC* American Joint Committee on Cancer

The results are described using number (%)/median (interquartile range), and statistically significant values (P < 0.05) are given in bold

Despite harvesting more lymph nodes (16 vs. 13, P = 0.008), there was no significant difference in the lymph node positivity rate between the LRAMPS and LDP groups (35.2% vs. 33.3%). Furthermore, the LRAMPS group demonstrated comparable rates of intravascular tumor thrombus (37.0% vs. 40.7%), perineural invasion (90.7% vs. 85.2%), adjacent tissue invasion (77.8% vs. 79.6%), R0 transection (94.4% vs. 96.3%), and retroperitoneal margin involvement (83.3% vs. 87.0%) when compared to the LDP group.

### Perioperative prognosis

The median postoperative length of stay was 10 days for both the LRAMPS and LDP groups. Furthermore, there were no significant differences observed in the incidence of complications based on the Clavien–Dindo classification between the two groups (Table 3).

**Table 3:** Perioperative and long-term prognosis in the PSM cohort

Variable	LDP (n = 54)	LRAMPS (n = 54)	P value
Postoperative length of stay, days	10 (8–12)	10 (8–13)	0.468
Clavien–Dindo classification			0.254
Grade 0–1	41 (75.9)	44 (81.5)	
Grade 2–3	13 (24.1)	10 (18.5)	
TAD on postoperative day 3, mL	40.0 (20.0–71.3)	85.0 (38.8–181.3)	**0.001**
DFA on postoperative day 3, U/L	5129.5 (2031.5–9929.5)	2802.0 (1204.0–6419.0)	**0.025**
POPF			0.761
None or biochemical	47 (87.0)	48 (88.9)	
Grade B	7 (13.0)	5 (9.3)	
Grade C	0 (0.0)	1 (1.9)	
Chyle leak, yes	3 (5.6)	8 (14.8)	0.202
Postpancreatectomy hemorrhage, yes	0 (0.0)	1 (1.9)	1.000
Delayed gastric emptying, yes	0 (0.0)	0 (0.0)	–
Postoperative transfusion, yes	1 (1.9)	2 (3.7)	1.000
Reoperation, yes	0 (0.0)	1 (1.9)	1.000
Semi liquid diet, days	4.0 (2.8–5.0)	5.0 (4.0–7.0)	**< 0.001**
Removal time of all abdominal drain, days	9.0 (7.0–16.0)	10.0 (7.0–13.0)	0.958
Readmission, yes	2 (3.7)	1 (1.9)	1.000
Death within 90 days, yes	0 (0.0)	0 (0.0)	–
Daily bowel movement frequency, > 2	3 (5.6)	10 (18.5)	**0.038**
Adjuvant chemotherapytherapy, yes	50 (92.6)	52 (96.3)	0.678
Adjuvant chemotherapy initiation time, weeks	5.9 (5.0–7.3)	6.2 (5.4–8.2)	0.156
Chemotherapy regimen			**0.010**
Combination chemotherapy based on gemcitabine	45 (90.0)	40 (76.9)	
Monotherapy with gemcitabine	4 (8.0)	2 (3.8)	
Oral chemotherapy (capecitabine or S-1)	1 (2.0)	10 (19.2)	
6-month intravenous chemotherapy completed^*^, yesDaily bowel movement frequency, > 2	41 (75.9)3 (5.6)	28 (51.9)10 (18.5)	**0.0160.038**

Recurrence pattern	LDP (n = 18)	LRAMPS (n = 21)	*P* value
Time of recurrence			0.757
Early recurrence	12 (66.7)	13 (61.9)	
Late recurrence	6 (33.3)	8 (38.1)	
Site of recurrence^**^			0.293
Local	7 (38.9)	5 (23.8)	
Liver	5 (27.8)	11 (52.4)	
Other distant metastasis	6 (33.3)	5 (23.8)	

*PSM* Propensity score matching, *LDP* laparoscopic distal pancreatosplecnectomy, *LRAMPS* laparoscopic radical antegrade modular pancreatosplenectomy, *TAD* total amount of abdominal drainage fluid, *DFA* abdominal drainage fluid amylase, *POPF* postoperative pancreatic fistula

The results are described using number (%)/median (interquartile range), and statistically significant values (P < 0.05) are given in bold

^*^The situation of incomplete 6-month postoperative intravenous chemotherapy includes: not receiving adjuvant chemotherapy due to physical reasons or personal choice; receiving oral chemotherapy only due to physical weakness; and switching to oral chemotherapy regimen due to intolerance to chemotherapy side effects during the intravenous chemotherapy process

^**^Defined as the site where tumor recurrence was first detected

The LRAMPS group exhibited a significantly higher median total amount of abdominal drainage fluid (TAD) on postoperative day 3 compared to the LDP group (85.0 vs. 40.0 mL, P = 0.001). However, the median levels of abdominal drainage fluid amylase (DFA) on postoperative day 3 were lower in the LRAMPS group compared to the LDP group (2802.0 vs. 5129.5 U/L, P = 0.025). In terms of pancreatic surgery-specific complications, there was no statistically significant difference observed in the incidence of POPF between the two groups. In the LRAMPS group, 8 patients (14.8%) experienced chyle leak, while in the LDP group, 3 patients (5.6%) had chyle leak, but this difference did not reach statistical significance. Notably, one patient in the LRAMPS group encountered mild early postoperative hemorrhage from a small vein on the posterior stomach wall. This complication was effectively resolved through laparoscopic exploration and hemostasis, and the patient was classified as having a grade C POPF and a Clavien–Dindo grade 3 complication. Furthermore, neither group experienced any cases of delayed gastric emptying or perioperative mortality.

The LRAMPS group exhibited a longer median time to resume a semi-liquid diet compared to the LDP group (5 vs. 4 days, P < 0.001). However, there were no statistically significant differences observed between the LRAMPS and LDP groups in terms of postoperative transfusion rate (3.7% vs. 1.9%), median time to removal of abdominal drain (10 vs. 9 days), and surgery-related readmission (1.9% vs. 3.7%).

### Long-term prognosis

**Fig. 3:**
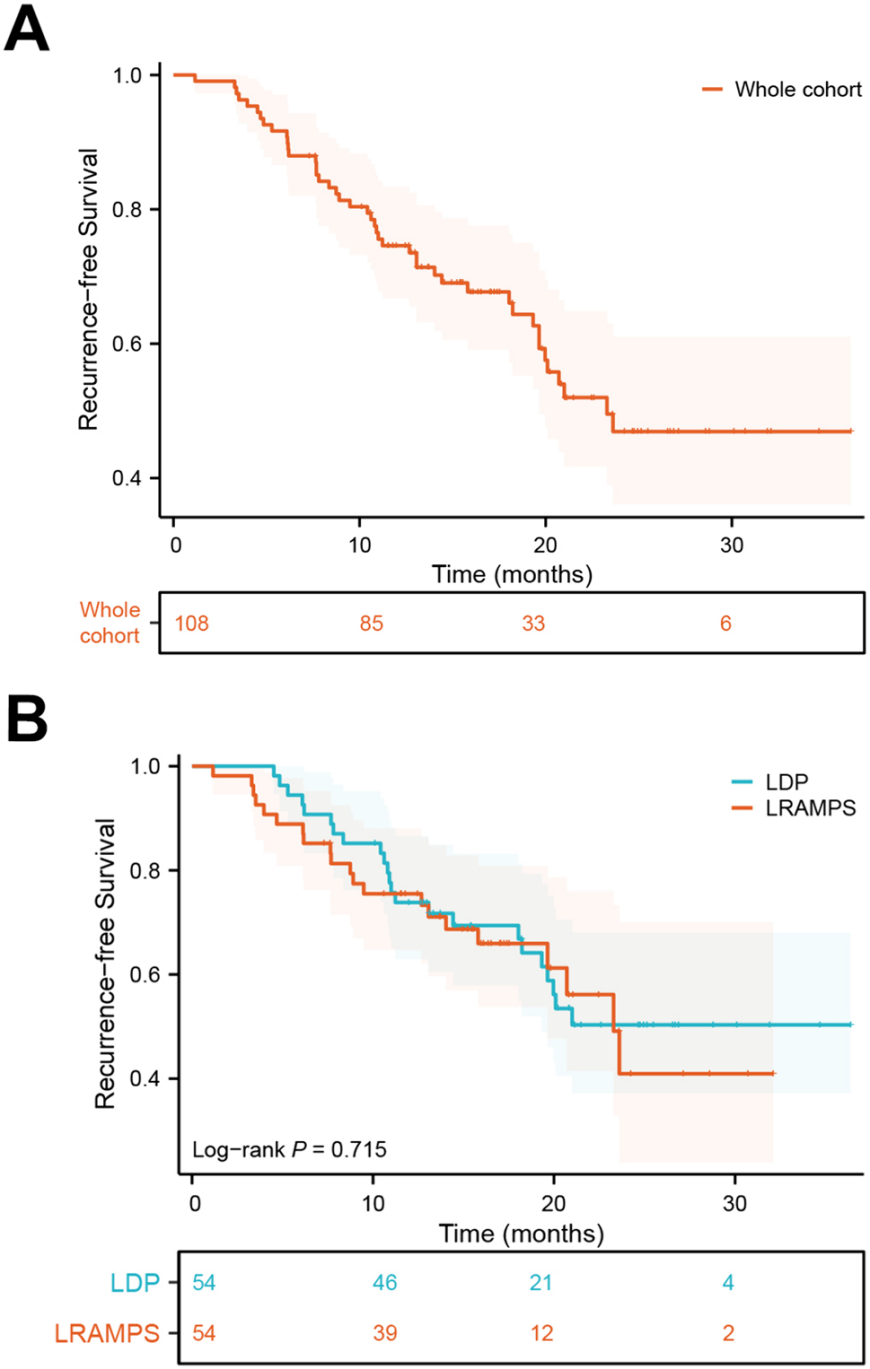
Recurrence-free survival for left-sided PDAC meeting the selection criteria. **A** Recurrence-free survival of the PSM cohort, **B** recurrence-free survival between LRAMPS and LDP patients. *PDAC* Pancreatic ductal adenocarcinoma, *LRAMPS* laparoscopic radical antegrade modular pancreatosplenectomy, *LDP* laparoscopic distal pancreatosplecnectomy

The median follow-up duration for this study was 20.4 months, and the median RFS was 23.3 months. There was no significant difference in RFS observed between the LRAMPS and LDP groups. The 6-month, 1-year, and 2-year RFS rates were 88.9%, 75.1%, and 41.6% for the LRAMPS group, and 94.4%, 73.7%, and 48.6% for the LDP group, respectively (P = 0.715, as illustrated in Fig. 3).

It is worth noting that the LRAMPS group had a higher proportion of patients with a daily bowel movement frequency greater than 2 compared to the LDP group (18.5% vs. 5.6%, P = 0.038). In terms of adjuvant chemotherapy, there was no statistically significant difference between the LRAMPS and LDP groups, with 96.3% and 92.6% of patients receiving it, respectively (P = 0.678). Although the median postoperative chemotherapy initiation time did not differ significantly (6.2 vs. 5.9 weeks, P = 0.156), the chemotherapy regimens significantly varied between the two groups. In the LRAMPS group, 76.9% received combination chemotherapy based on gemcitabine, 3.8% received gemcitabine monotherapy, and 19.2% received oral chemotherapy (capecitabine or S-1). In contrast, the corresponding proportions in the LDP group were 90.0%, 8.0%, and 2.0% (P = 0.010). Furthermore, completion of the 6-month intravenous chemotherapy was lower in the LRAMPS group compared to the LDP group (51.9% vs. 75.9%, P = 0.016) (Table 3).

Among the 39 patients who experienced tumor recurrence, 21 belonged to the LRAMPS group, while 18 were from the LDP group. There was no significant difference observed in the time to recurrence between the two groups. The rates of early recurrence were 61.9% in the LRAMPS group and 66.7% in the LDP group (P = 0.757). The site of first recurrence did not significantly differ between the groups. In the LRAMPS group, the proportions of local recurrence, liver metastasis, and other distant metastasis were 23.8%, 52.4%, and 23.8%, respectively. In the LDP group, the corresponding proportions were 38.9%, 27.8%, and 33.3% (P = 0.293).

## Discussion

This PSM cohort study revealed that LRAMPS did not offer oncological advantages over LDP for left-sided PDAC within the specified selection criteria. The LRAMPS procedure did not yield a higher number of positive lymph nodes or a more extensive margin-negative rate. On the contrary, it resulted in longer operation time, increased intraoperative bleeding and postoperative abdominal drainage volume. It also led to a prolonged recovery period for gastrointestinal function and an increase in postoperative bowel movement frequency. These factors can impact the regimen selection and completion of postoperative adjuvant chemotherapy, consequently compromising the potential benefits of LRAMPS in achieving better local control. The study suggests that LDP is sufficient to meet the oncologic requirements for selected left-sided PDAC.

Due to the resection plane of the RAMPS procedure being located behind Gerota’s fascia, as opposed to the traditional distal pancreatosplecnectomy resection plane which is located behind the pancreatic capsule, it significantly increases the number of N1 lymph nodes harvested and improves the R0 rate of the posterior tangential margin of the specime [5–7]. The study conducted by Grossman et al. included 78 cases of RAMPS, with 93.6% of patients achieving negative tangential margins. The average number of lymph nodes retrieved was 20, with an average of 1.4 positive lymph nodes [26]. Meta-analyses further support the superiority of RAMPS over standard procedures, demonstrating improved R0 resection rates and lymph node retrieval rates [27, 28]. Recent studies have shown that LRAMPS is technically feasible and provides comparable long-term oncological outcomes to open RMAPS [10, 29]. Additionally, LRAMPS demonstrates similar rates of postoperative complications to open RMAPS but also exhibits lower intraoperative blood loss and faster postoperative recovery of eating time [9]. Given the oncological equivalence of LRAMPS and its advantages in short-term outcomes, several studies have examined the feasibility of LRAMPS as the standard treatment for resectable left-sided PDAC [13–15].

However, in previous studies focusing on the oncology and safety of RAMPS, most of the tumors analyzed were at T2-stage or above [8, 12, 13]. For tumors within T2 stage and confined to the pancreatic parenchyma, the necessity of expanding the resection extent and dissecting more N1 lymph nodes requires further exploration. Referring to the Korean LRAMPS Yonsei criteria [12], we proposed our selection criteria and conducted this PSM cohort analysis. Although the LRAMPS group retrieved more lymph nodes compared to the LDP group (16.0 vs. 13.0, P = 0.008), this did not result in a higher detection rate of positive lymph nodes (35.2% vs. 33.3%, P = 0.839). Recent studies on anti-tumor immunity of solid tumors have found that uninvolved lymph nodes are enriched with progenitor exhausted CD8 + T cells, while the anti-tumor immune hallmarks were impaired in metastatic lymph nodes and exhibited immunosuppressive cellular niches [30]. Therefore, removing too many negative lymph nodes, particularly for immunologically “cold” tumors like PDAC, may potentially worsen the immune microenvironment. Moreover, LRAMPS did not increase the detection rate of positive retroperitoneal margins in PDAC cases meeting the selection criteria (16.7% vs. 13.0%, P = 0.588). But, it did result in longer operation time (240.5 vs. 219.0 min, P = 0.020) and increased intraoperative blood loss (200 vs. 150 mL, P = 0.001). However, given that most left-sided PDACs are discovered at a stage greater than T2 and invade the fascial layer behind the pancreas in clinical practice, RAMPS remains the primary approach for achieving oncological resection in left-sided PDAC cases beyond our proposed selection criteria.

A study conducted in the United States analyzed data from the National Surgical Quality Improvement Project database, comparing 236 distal pancreatosplecnectomy patients with 117 RAMPS patients. The study found that RAMPS had comparable surgical safety to distal pancreatosplecnectomy, as it did not increase postoperative complications such as overall POPF, clinically relevant POPF, and Clavien–Dindo grade 3 or higher complications [14]. However, it is important to note that in this study, the RAMPS group had a low proportion of minimally invasive surgery, accounting for only 6.8% of cases. Currently, there is limited research comparing LRAMPS and LDP [31]. In our study, we found no significant difference in the length of postoperative hospital stay between the two groups, with a median hospital stay of 10 days after surgery. It is understandable that LRAMPS, with its larger resection extent, would result in a higher postoperative TAD compared to LDP (85.0 vs. 40.0 mL, P = 0.001), which in turn leads to dilution of the DFA (2802.0 vs. 5129.5 U/L, P = 0.025). Moreover, although not reaching statistical significance, the incidence of chyle leak in the LRAMPS group appears to be higher than that in the LDP group (14.8% vs. 5.6%). We believe that with an increased sample size, statistical significance may be achieved. However, these differences did not result in a significant difference in the occurrence of POPF between the two groups. While one patient in the LRAMPS group experienced early mild hemorrhage that required reoperation for hemostasis and was classified as having a grade C POPF and a Clavien–Dindo grade 3 complication, there were no statistically significant differences between the two groups in terms of delayed gastric emptying, and no deaths occurred within 90 days of surgery in either group.

However, it should be noted that LRAMPS did prolong the time required for patients to recover to a semi-liquid diet after surgery. Additionally, due to the removal of more nerve plexus during LRAMPS [16], there was an increase in daily bowel movement frequency after surgery, which could potentially impact the patients' quality of life. The impact of LRAMPS surgery on patients' physical recovery can also be observed in the timing of initiation, regimen selection, and completion of adjuvant chemotherapy. It appears that patients in the LRAMPS group started adjuvant chemotherapy later, although this difference did not reach statistical significance. However, in the LDP group, a higher proportion of patients received intravenous chemotherapy (98.0% vs. 80.7%) and had a higher completion rate of 6-month intravenous chemotherapy (75.9% vs. 51.9%). Therefore, when evaluating the potential oncological benefits of LRAMPS surgery, it is crucial to fully consider the impact of this technique on overall treatment outcomes.

To date, there is no evidence to suggest that RAMPS surgery improves postoperative RFS or overall survival. However, it may be effective in controlling local tumor recurrence. A study conducted in Japan analyzed the clinical data of 174 patients who underwent open distal pancreatosplecnectomy and open RAMPS between 2009 and 2016. Following PSM analysis, the study found no significant difference in 3-year RFS and overall survival between the two groups. However, the open RAMPS group did demonstrate a lower 3-year local recurrence rate compared to the open distal pancreatosplecnectomy group (10% vs. 34%, hazard ratio = 0.275, P = 0.024) [8]. Our own study on LRAMPS compared to LDP found no RFS benefit. Among the PSM cohort, a total of 39 patients experienced tumor recurrence, with 21 patients in the LRAMPS group and 18 patients in the LDP group. We further examined the recurrence patterns between the two groups. Consistent with the Japanese study, LRAMPS, with its wider resection range, showed potential for improved local control. This was evident in the lower rates of early recurrence (61.9% vs. 66.7%) and local recurrence (23.8% vs. 38.9%) in the LRAMPS group compared to the LDP group. However, these differences were not statistically significant. Nevertheless, LRAMPS had an impact on postoperative physical recovery, leading to a lower completion rate of adjuvant intravenous chemotherapy. This significantly compromised the potential benefits of better local control. Overall, our study found that, for selected early-stage left-sided PDAC, LRAMPS did not provide oncological advantage over LDP.

Our study is the first to discuss the necessity of LRAMPS surgery for selected early-stage left-sided PDAC, but it still has some limitations. Firstly, although we utilized PSM to minimize intergroup bias, we were unable to achieve the effects of a randomized controlled clinical trial. Secondly, although all surgeons involved in this study had surpassed their learning curve for LRAMPS, it is important to note that this study was conducted at a high-volume center in China, which may impact the generalizability of the results to other centers. Finally, the follow-up duration in this study was relatively short, and longer follow-up is necessary to observe the overall survival impact of LRAMPS on left-sided PDAC within the specified selection criteria. However, we believe that our study offers valuable insights into the application of LRAMPS in early-stage resectable left-sided PDAC.

## Conclusions

Our study revealed that LRAMPS did not offer oncological advantages over LDP for selected early-stage left-sided PDAC. In fact, it led to longer operation time, increased intraoperative bleeding, prolonged recovery time for gastrointestinal function, and impacted the postoperative daily bowel movement frequency of patients. Additionally, it also influenced the regimen selection and completion of postoperative adjuvant chemotherapy, thereby compromising the potential benefits of LRAMPS in achieving better local control. These findings suggest that LDP is adequate in meeting the oncologic requirements for left-sided PDAC within our specified selection criteria. However, further prospective, randomized controlled trials with larger sample sizes and longer follow-up periods are necessary to validate these findings.

### Supplementary Information

Below is the link to the electronic supplementary material.

Supplementary file1 (DOCX 40 kb)
