# Supplementary material for: Evaluating the efficacy of laparoscopic radical antegrade modular pancreatosplenectomy in selected early-stage left-sided pancreatic cancer: a propensity score matching study
Source: Surg Endosc. 2024 May 15;38(7):3578–89. doi: 10.1007/s00464-024-10868-x (PMC11219433; doi:10.1007/s00464-024-10868-x)
Supplement: Supplementary file 1 — Supplementary file1 (DOCX 40 kb) [file 464_2024_10868_MOESM1_ESM.docx]

| **Supplementary Table 1. Logistic regression analysis of confounding variables between the LDP group and the LRAMPS group (n=185)** | | | |
| --- | --- | --- | --- |
| **Variable** | **OR** | **95% CI** | ***P* value** |
| **Age**, years (continuous) | 1.000 | 0.966-1.036 | 0.982 |
| **Gender**, male vs. female | 0.916 | 0.482-1.741 | 0.788 |
| **Body mass index**, kg/m^2^ (continuous) | 0.960 | 0.851-1.084 | 0.512 |
| **Diabetes mellitus**, yes vs. no | 1.549 | 0.755-3.176 | 0.232 |
| **Hypertension**, yes vs. no | 0.642 | 0.333-1.238 | 0.186 |
| **History of abdominal surgery**, yes vs. no | 0.791 | 0.365-1.714 | 0.553 |
| **White blood cell**, 10^^^9/L (continuous) | 1.020 | 0.917-1.134 | 0.720 |
| **Hemoglobin**, g/L (continuous) | 1.003 | 0.983-1.023 | 0.779 |
| **Albumin**, g/L (continuous) | 0.956 | 0.866-1.055 | 0.370 |
| **Total bilirubin**, μmol/L (continuous) | 0.992 | 0.939-1.049 | 0.786 |
| **Serum creatinine**, μmol/L (continuous) | 0.989 | 0.966-1.011 | 0.325 |
| **Prothrombin time**, seconds (continuous) | 1.080 | 0.840-1.388 | 0.548 |
| **CA19-9**, U/mL (continuous) | 0.999 | 0.998-1.000 | 0.250 |
| **Tumor diameter on imaging**, cm (continuous) | 1.495 | 1.002-2.230 | **0.049** |
| **Tumor differentiation**, poor vs. moderate/well | 2.310 | 1.211-4.405 | **0.011** |

Abbreviation: LDP, laparoscopic distal pancreatosplecnectomy; LRAMPS, laparoscopic radical antegrade modular pancreatosplenectomy; CA19-9, carbohydrate antigens 19-9.
